# Allelic variation of carotenoid biosynthesis and degradation genes across worldwide commercial durum wheat (*Triticum turgidum* ssp. *durum*) varieties under contrasting water regimes

**DOI:** 10.1007/s11032-026-01641-0

**Published:** 2026-02-18

**Authors:** Virginia Garcia-Calabres, Juan B. Alvarez, Maria Itria Ibba, Nayelli Hernández-Espinosa, Karim Ammar, Carlos Guzmán

**Affiliations:** 1 Departamento de Genética, Escuela Técnica Superior de Ingeniería Agronómica y de Montes, Edificio Gregor Mendel, Campus de Rabanales, Universidad de Córdoba, CeiA3, ES-14071 , Córdoba, Spain; 2 Global Wheat Program, International Maize and Wheat Improvement Center (CIMMYT), Apdo Postal 6-641, Mexico, DF Mexico

**Keywords:** Durum wheat, Yellow pigment content, Lipoxygenases, Marker assisted selection

## Abstract

Carotenoids are the pigments responsible for the yellow colour of durum wheat semolina, a key quality trait for pasta production. This study evaluated 46 commercial durum wheat cultivars grown under irrigated and drought-simulated conditions in the Yaqui Valley (Mexico), measuring yellow pigment content (YPC) and yellow pigment loss (YPL) under simulated processing conditions. Cultivars under the full irrigation system increased YPC (7.3 mg/kg) and reduced YPL (13.3%) compared to cultivars under drip irrigation system or simulated drought (YPC 6.6 mg/kg; YPL 15.3%), indicating that probably water limitation reduces carotenoid accumulation and enhances pigment degradation. Allelic variation in carotenoid biosynthesis genes (*PSY-A1*, *PSY-B1*, *PDS-B1*, *ZDS-A1*) and degradation genes (*LPX-A3*, *LPX-B1.1*) was assessed. *Psy-A1o*, *Psy-B1n*, and *TdZds-A1.2* alleles were associated with higher YPC, while *Lpx-B1.1c* contributed to lower pigment loss. No significant effects were observed for *PDS-B1* or *LPX-A3*, and no cultivar carried all favourable alleles simultaneously, highlighting the potential for further improvement and pyramiding of carotenoid-related traits. This study provides molecular markers and genotype information for breeding programs aimed at improving semolina and pasta colour, offering strategies to enhance pigment retention under contrasting water regimes.

**Supplementary Information:**

The online version contains supplementary material available at 10.1007/s11032-026-01641-0.

## Introduction

Durum wheat (*Triticum turgidum* ssp. *durum* Desf. em. Husn.) is the raw material to produce semolina, pasta, and numerous traditional products in the Mediterranean Basin (Faridi and Faubion 1995). Its preference over common wheat (*T. aestivum* L. ssp. *aestivum*) stems from its suitability for semolina milling and gluten properties that ensure good cooking quality and resistance to overcooking in pasta products (Sissons 2008). Additionally, durum wheat is valued for its characteristic bright yellow colour, which is associated with increased consumer acceptance (Borrelli et al. 2003; Randhawa et al. 2013).

Carotenoids are the compounds responsible for this yellow pigmentation. The carotenoid biosynthetic pathway begins with the production of phytoene, a reaction catalysed by phytoene synthase (PSY). This is followed by a series of desaturation and isomerization steps, catalysed by various enzymes among which phytoene desaturase (PDS) and ζ-carotene desaturase (ZDS) stand out, leading to the biosynthesis of lycopene. Further lycopene modifications generate mainly lutein (86–94%), along with small amounts of zeaxanthin, β-cryptoxanthin, α-carotene and β-carotene (Adom et al. 2003; Hentschel et al. 2002). Lutein acts as a blue light filter, protecting retinal structures (Kijlstra et al. 2012), and its intake has been associated with benefits against eye diseases such as cataracts and age-related macular degeneration (Buscemi et al. 2018; Sawa et al. 2020). Positive effects on inflammatory responses, neuroprotection, and the reduction of coronary and systemic disorders have also been described (Mitra et al. 2021).

Breeding for increased carotenoid content is essential, given consumer preferences and the increasingly restrictive legislation on the use of artificial colorants in pasta production. Some countries, such as Italy, France or Greece, mandate that dry pasta must be produced exclusively from durum wheat semolina with no colorants added (Sissons 2008). Furthermore, enhancing carotenoid levels is particularly important in developing countries, where the availability of these bioactive compounds is limited.

Nevertheless, pasta yellowness does not depend solely on the endogenous carotenoid content. The final colour of the end-products is also influenced by semolina yield, processing conditions, and oxidative degradation – primarily caused by three groups of enzymes: peroxidases, polyphenol oxidases and lipoxygenases (LPX), the latter being the main contributors to colour degradation (Borrelli et al., 2016; Colasuonno et al. 2019). Consequently, the development of functional molecular markers has become essential to pyramid favourable alleles for both high biosynthesis and low degradation. Available tools now include markers for genes in the biosynthetic pathway, such as *PSY* (Singh et al. 2009), *PDS* (Pasten et al. 2021), and *ZDS* (Rasheed et al. 2016), alongside markers for alleles conferring reduced lipoxygenase activity (Carrera et al. 2007).

Despite being a highly heritable trait, carotenoid concentration in durum wheat can be influenced by various environmental factors, such as water deficit or sulphur fertilization (Fratianni et al. 2013). In the Mediterranean Basin and the Middle East, durum wheat is grown primarily under rainfed conditions, making drought stress one of the most common abiotic stresses. In contrast, in regions such as California in USA or Sonora in Mexico, irrigation is used to ensure consistent production. Therefore, understanding how water availability affects quality characteristics, such as yellow pigment biosynthesis and degradation, is important for breeding programs.

This study was conducted to: (i) evaluate the effects of irrigation and drought stress on yellow pigment content (YPC) and yellow pigment loss (YPL); (ii) assess the genotypic variation associated with YPC and YPL; and (iii) investigate the relationship between allelic variation in key genes and their influence on YPC and YPL.

## Materials and methods

### Plant material

Forty-six durum wheat cultivars, representing popular commercial cultivars grown in 12 different countries, were used in this study. These cultivars were grown at the Campo Experimental Norman E. Borlaug (CENEB), near Ciudad Obregón (Sonora, Mexico) during the 2014/15 cropping season. The genotypes were sown in November in 1m^2^ size plots and harvested in May. A randomized complete block design with three replicates was used, under two different water regimes: fully irrigated (550 mm) and drip irrigation simulating drought conditions (180 mm). Weed control, disease management, and insect control were rigorously maintained. Nitrogen (N) was applied at a rate of 50 kg/ha of N (50 N units) prior to planting. Additionally, at tillering 150 N units were applied for the irrigated conditions and 50 N units for the drought conditions. The amount of nitrogen applied was sufficient to ensure that nitrogen was not a limiting factor in the study. At maturity, entire plots were mechanically harvested.

During the experiment, the meteorological conditions were characterized by almost no precipitation throughout the wheat growing season, ensuring the reliability of the drought treatment (total rain of 17 mm from January to April). Maximum temperatures ranged from 30 to 35 °C in March and April, during the grain filling period for both water regimes. Weather data for temperature and rainfall were obtained from a local weather station located 2 km from CENEB (Sistema de Alerta Fitosanitaria del Estado de Sonora [SIAFESON], 2025).

The mean grain yield in the fully irrigated plots was 5.0 t/ha, whereas drip-irrigated plots yielded 2.6 t/ha, confirming the effectiveness of the drought treatment. Grain samples of 800 g were stored in a storage room with stable temperature (15 °C) and used for quality analysis five months after the harvest. The grain samples were evaluated for moisture content by near-infrared spectroscopy (DA7200 NIR, PerkinElmer Inc., Waltham, MA, USA) following the official AACC method 44-15.02 (American Association of Cereal Chemists [AACC], 2010). Before milling, the grain samples were conditioned to 16% moisture content for 48 h and then milled using a Brabender Quadrumat Jr. (C.W. Brabender OHG, Germany) to obtain flour.

### Yellow pigment content and yellow pigment loss

Two days after the milling, the yellow pigment content (YPC, mg carotenoids/kg flour) was assessed using the micro-scale semolina pigment content assay developed by Fu et al. (2013), based on the AACC approved method 14-50.01 (AACC, 2010) with flour used instead of semolina. This parameter was measured both in the flour and in a dough formed to simulate oxidative conditions that occur during pasta processing. Yellow Pigment Loss (YPL, %) was calculated as the percentage difference between the YPC measured in the flour and that measured in the formed dough.

Two samples of 200 ± 5 mg of flour were weighed and placed into 2 mL microfuge tubes. One sample was mixed with 1 mL of water-saturated 1-butanol (85:15) and homogenized using a vortex. The other sample was mixed with 0.15 mL of water and homogenized using a metal spatula to form a dough. The resulting dough was left to rest for 2 h with the lid open at room temperature, under continuous airflow and light within a laminar flow cabinet. The YPC of the rested dough was then extracted by adding 0.85 mL of pure 1-butanol, followed by homogenization with the metal spatula after 20 min.

For both samples, after resting for 1 h, the mixture was centrifuged at 15,000 *g* for 10 min, and 300 µl of the supernatant was transferred to a 96-well microplate. Absorbance was measured at 436 nm using a SPECTROstar NANO^®^ spectrophotometer (BMG Labtech, Ortenberg, Germany). The YPC was measured in duplicate for all samples and determined based on a calibration curve generated with known quantities of lutein.

### Molecular marker analysis

Genomic DNA was extracted from the leaf tissues of 4–5 young seedlings per genotype using the CTAB method (Stacey et al., 1994). Molecular marker analysis was performed for the genes *PSY-A1*, *PSY-B1*, *PDS-B1*, *ZDS-A1*, *LPX-A3* and *LPX-B1.1*. PCR reactions were performed in a final volume of 20 µl containing 50 ng of genomic DNA, 1× PCR buffer, MgCl₂ at a final concentration of 1.5 mM, dNTPs at 0.2 mM, 0.2 µM of each primer (forward and reverse), 1 U of Taq DNA polymerase, and nuclease-free water. The PCR conditions, the list of primers and their sequences, were established following the indications described by Garcia-Calabres et al. (2025). The enzymes *Hae*II (New England Biolabs) and *HpyCH4*IV (New England Biolabs) were used to digest the PCR products of the *LPX-A3* and *PDS-B1* genes, respectively. Digestion was performed at 37 °C for 2 h in both cases.

The PCR products and digestion fragments were electrophoretically separated by polyacrylamide gel electrophoresis (PAGE) under the following conditions: 10% (w/v, C: 1.28%) were used in all cases, except for *ZDS-A1*, which was run on a 12% (w/v, C: 0.44%) gel, and *LPX-A3*, which was run on an 8% (w/v, C: 1.28%) gel. Bands were stained with GelRed™ Nucleic Acid Stain (Biotium) and visualized under UV light.

### Statistical analyses

Data were analysed using Type III analysis of variance (ANOVA), considering genotype, water regime, and their interaction as sources of variation. The water regime included both irrigated and drought conditions. Mean comparisons were performed using estimated marginal means (emmeans) obtained from the linear model, followed by Tukey’s HSD test at *P* < 0.05.

To investigate the relationship between allelic variation and its influence on YPC and YPL, Type III ANOVAs were conducted using key carotenoid biosynthesis genes (*PSY-A1*, *PSY-B1*, *PDS-B1*, and *ZDS-A1*) and lipoxygenase genes (*LPX-A3* and *LPX-B1.1*) as factors, respectively. Interactions among genes were excluded from the model due to insufficient representation of all possible allelic combinations. Emmeans were compared using Tukey’s HSD test at *P* < 0.05.

Significant differences among means are indicated by superscript letters in figures and tables. The assignment of letters reflects the direction of interest for each trait: for YPC, the letter “a” denotes the highest (most desirable) value, whereas for YPL, “a” indicates the lowest (most favourable) value. Subsequent letters represent statistically decreasing (YPC) or increasing (YPL) values relative to the optimum.

All statistical analyses were performed using RStudio (version 2024.12.0 + 467, Posit, PBC, Boston, MA, USA).

## Results

### Effects of cultivar, water regime and their interaction on YPC and YPL

All data related to this study are available in Supplementary Table S1. The effects of genotype, water regime, and their interaction on YPC and YPL are provided in Supplementary Table S2. Although the genotype was the main source of variation for both YPC and YPL, the water regime and the genotype × water regime interaction were also highly significant.

**Fig. 1:**
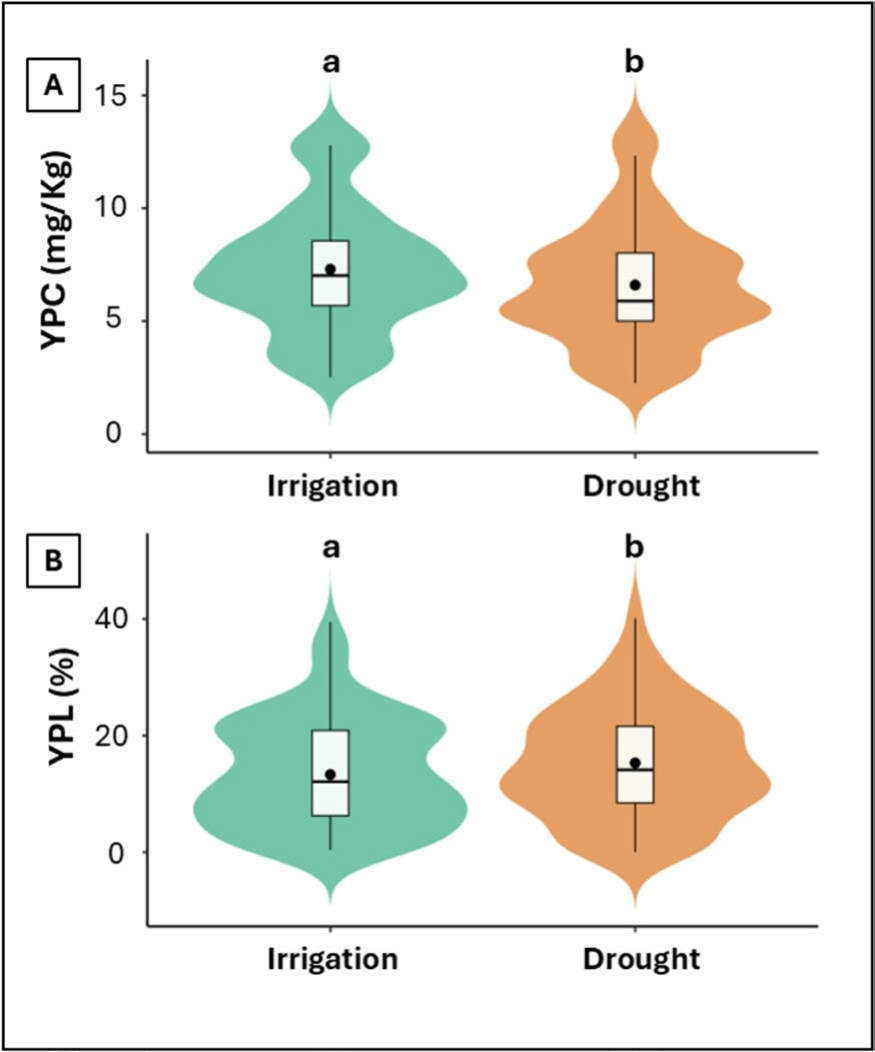
**A**) Yellow pigment content (YPC, mg carotenoids/kg flour) and **B**) yellow pigment loss (YPL, %) of 46 durum wheat cultivars grown under irrigated and drought conditions. Violin plots with boxplots show data distribution, points represent emmeans. *Different letters indicate significant differences according to Tukey’s HSD test* (*P < 0.05)*

The distribution of YPC and YPL under simulated processing conditions for both irrigated and drought conditions is shown in Fig. 1. For YPC, carotenoid content in flour ranged from 2.5 to 13.6 mg carotenoids/kg flour, with a mean of 7.3 mg/kg under irrigation (Fig. 1A). Under drought conditions, the range was slightly lower (2.3–13.5 mg/kg) with a mean of 6.6 mg/kg. Median YPC was also higher under irrigation (7.0 mg/kg) than under drought conditions (5.9 mg/kg). For most cultivars (38 of 46), YPC decreased under drought relative to irrigation, whereas eight cultivars exhibited the opposite trend.

The impact of water regime on YPL was similarly evaluated (Fig. 1B). Under irrigated conditions, YPL ranged from 0.4% to 39.5%, with a mean of 13.3%. Drought conditions led to greater pigment degradation, with a broader range (0.0–41.6%) and a higher mean of 15.3%. Median YPL was 12.1% under irrigation and 14.1% under drought, indicating that drought conditions tend to increase pigment loss. Most cultivars (29 of 46) had higher YPL under drought than under irrigation, whereas 17 cultivars showed the opposite trend.

**Fig. 2:**
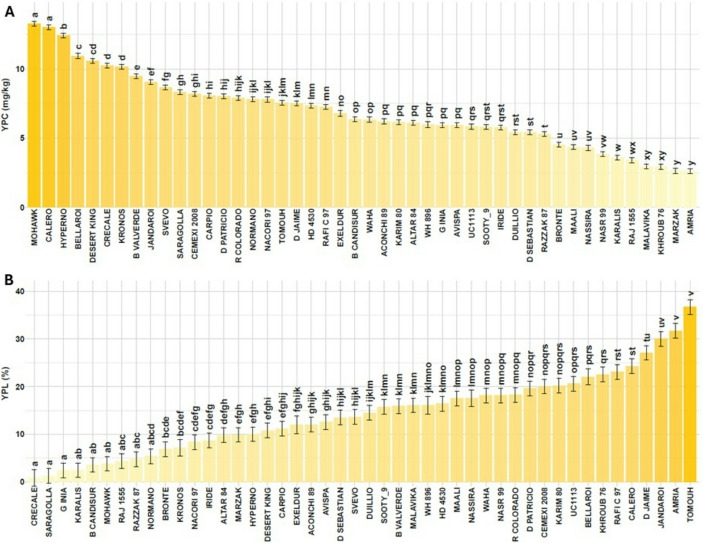
**A**) Yellow pigment content (YPC, mg carotenoids/kg flour) and **B**) yellow pigment loss (YPL, %) of 46 durum wheat genotypes. Bars represent emmean values ± standard error (SE). *Different letters indicate significant differences according to Tukey’s HSD test (P < 0.05)*

Figure 2A presents the average YPC for 46 commercial durum wheat cultivars under both conditions. The group with the highest YPC group included the desert durum Mohawk and the Spanish cultivar Calero (~ 13 mg/kg), followed by three sub-groups with values above 10 mg/kg, including Australian cultivars (Hyperno and Bellaroi), some additional desert durum wheats (Desert King and Kronos) and the Italian cultivar Grecale. At the lower end of the spectrum (YPC < 3 mg/kg) were North African cultivars, mostly of CIMMYT origin from the late 1970s and early 1980s, such as Marzak and Amria (Morocco), Khroub 76 (Algeria) and Malavika and Raj 1555 (India).

Regarding YPL (Fig. 2B), nine cultivars exhibited low pigment degradation (0.8–5.4%), forming the first significance group. The Italian cultivar Grecale, which also had high YPC, displayed the lowest YPL. Other cultivars in this group included both high YPC (e.g., Mohawk) and low YPC (Karalis from Italy, Razzak from Tunisia and Raj 1555) as well as cultivars with intermediate YPC. Cultivars with the greatest pigment losses included both high YPC (Calero and the Australian cultivar Jandaroi) and low YPC (Amria and Tomouh from Morocco).

### Allelic variation in carotenoid biosynthesis and degradation genes

Allelic variation was analysed in three genes involved in carotenoid biosynthesis (*PSY-1*,* PDS-B1* and *ZDS-A1*) and two genes associated with carotenoid degradation (*LPX-A3* and *LPX-B1.1*). The key gene in the biosynthetic pathway, *PSY-1*, is present in both genomes of durum wheat as *PSY-A1* and *PSY-B1*. Three alleles were identified for *PSY-A1* gene: *Psy-A1a*, *Psy-A1l*, and *Psy-A1o*. *Psy-A1l* allele was the most frequent allele, detected in 34 of the 46 cultivars, followed by *Psy-A1a* (*n* = 9) and *Psy-A1o* (*n* = 3). Two alleles were identified for *PSY-B1*: *Psy-B1o*, present in forty genotypes, while six cultivars carried the *Psy-B1n* allele. For the *PDS-B1* gene, encoding the next enzyme in the carotenoid biosynthetic pathway, all cultivars carried the *Pds-B1b* allele except for Atil C2000, which carried the *Pds-B1a* allele. Finally, with regards to the *ZDS-A1* gene, two variants were identified: *TdZds-A1.1* and *TdZds-A1.2.* The *TdZds-A1.1* allele was present in most cultivars, except for Desert King and Cemexi C 2008 that exhibited the *TdZds-A1.2* allele.

Regarding carotenoid degradation, *LPX-A3* showed two alleles, with thirty cultivars carrying the *UC1113 Lpx-A3* allele, and sixteen cultivars presenting the *Kofa Lpx-A3* allele. The difference between both alleles is related with the digestion of the 920-pb amplicon by the enzyme *HaeII* for the *UC1113* allele, whereas this amplicon was not digested in the case of *Kofa* allele (Carrera et al. 2007). For the *LPX-B1.1* gene, three alleles were detected: *Lpx-B1.1a*, *Lpx-B1.1b* and *Lpx-B1.1c* (deletion of the gene). Thirty-one cultivars presented the *Lpx-B1.1a* allele, fourteen the *Lpx-B1.1c* allele and only the cultivar Tomouh presented the *Lpx-B1.1b* allele.

The 46 cultivars were classified based on allelic combinations in the key genes associated with carotenoid biosynthesis and degradation. Table 1 lists the alleles identified for each gene in all 46 commercial durum wheat cultivars included in the study. Based on this variation, the cultivars were grouped into sixteen distinct haplotypes, which were ordered from I to XVI based on their YPC from highest to lowest. The most frequent haplotypes were XI, VIII, X and XIV, represented by 9, 8, 6 and 6 cultivars, respectively.

**Table 1:** Allelic combination groups for the carotenoid synthesis genes *PSY-A1*, *PSY-B1*, *PDS-B1*, *ZDS-A1* and degradation genes *LPX-A3*, *LPX-B1.1* in durum wheat cultivars evaluated

Haplotype	PSY-A1	PSY-B1	PDS-B1	ZDS-A1	LPX-A3	LPX-B1.1	YPC ± SE	YPL ± SE	Cultivar
I	*l*	*n*	*b*	*A1.1*	*UC1113*	*c*	11.7 ± 0.5 a	5.4 ± 2.0 ab	Kronos, Mohawk
II	*o*	*o*	*b*	*A1.1*	*Kofa*	*a*	11.2 ± 0.8 abc	22.2 ± 3.0 def	Bellaroi
III	*o*	*o*	*b*	*A1.2*	*UC1113*	*c*	10.6 ± 0.8 abc	10.8 ± 2.8 abcde	Desert King
IV	*l*	*n*	*b*	*A1.1*	*UC1113*	*a*	10.5 ± 0.5 ab	17.7 ± 2.0 cde	Calero, Carpio
V	*o*	*n*	*b*	*A1.1*	*UC1113*	*a*	9.0 ± 0.8 abcd	30.0 ± 2.8 fg	Jandaroi
VI	*l*	*o*	*b*	*A1.2*	*Kofa*	*a*	8.2 ± 0.8 bcde	20.0 ± 2.8 cdef	Cemexi C 2008
VII	*l*	*o*	*b*	*A1.1*	*Kofa*	*b*	7.6 ± 0.8 bcdef	36.7 ± 2.8 g	Tomouh
VIII	*l*	*o*	*b*	*A1.1*	*Kofa*	*a*	7.6 ± 0.3 d	14.5 ± 1.0 cd	Aconchi 89, Altar 84, Bonaerense Valverde, Don Patricio, Hyperno, Malavika, Rafi C 97, Rio Colorado
IX	*l*	*n*	*b*	*A1.1*	*Kofa*	*a*	7.3 ± cdef	16.4 ± 2.8 bcde	HD 4530
X	*l*	*o*	*b*	*A1.1*	*UC1113*	*c*	7.1 ± 0.3 de	5.9 ± 1.1 a	Buck Candisur, Grecale, Guayacan Inia, Nacori C 97, Nasr 99, Saragolla
XI	*l*	*o*	*b*	*A1.1*	*UC1113*	*a*	6.3 ± 0.2 de	14.8 ± 0.9 cd	Avispa, Don Jaime, Don Sebastián, Exeldur, Iride, Karalis, Svevo, Waha, WH 896
XII	*l*	*o*	*a*	*A1.1*	*Kofa*	*a*	5.8 ± 0.8 defg	15.8 ± 2.8 abcde	Atil C2000
XIII	*l*	*o*	*b*	*A1.1*	*Kofa*	*c*	5.5 ± 0.4 efg	4.8 ± 1.6 a	Normano, Raj 1555, Razzak 87
XIV	*a*	*o*	*b*	*A1.1*	*UC1113*	*a*	4.8 ± 0.3 fg	20.6 ± 1.1 ef	Amria, Duillio, Karim 80, Maali, Nassira, UC1113
XV	*a*	*o*	*b*	*A1.1*	*UC1113*	*c*	3.6 ± 0.5 g	8.4 ± 2.0 abc	Bronte, Marzak
XVI	*a*	*o*	*b*	*A1.1*	*Kofa*	*a*	2.9 ± 0.8 g	22.2 ± 3.0 def	Khroub 76

YPC (mg/kg): Yellow Pigment Content; YPL (%): Yellow Pigment Loss; SE: Standard Error. *Values followed by different letters are significantly different according to Tukey ’s HSD test *(*P *< 0.05)

### Genetic association for yellow pigment content and yellow pigment loss

For these analyses, data from both environments were combined to obtain an integrated assessment of allelic effects while accounting for environmental variability. Averaging the results across water regimes reduced environment-specific fluctuations and highlighted genetic effects that were stable and consistent across contrasting growing conditions.

**Fig. 3:**
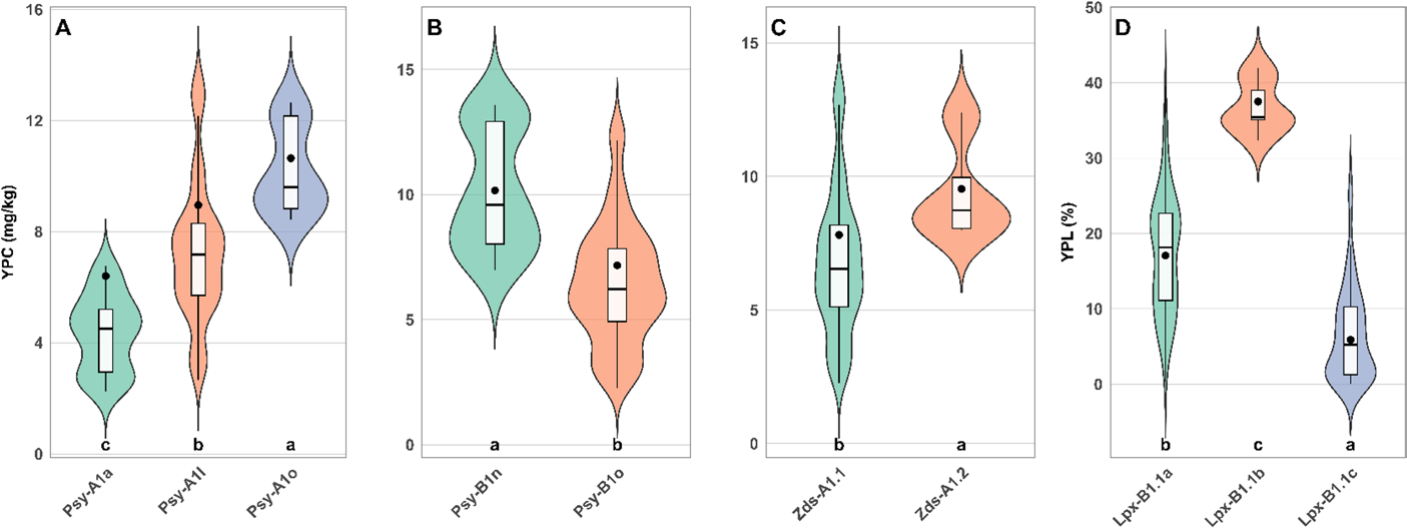
Yellow pigment content (YPC, mg carotenoids/kg flour) and yellow pigment loss (YPL, %) by allelic variation at significant carotenoid biosynthesis and degradation genes. Panels show **A**) *PSY-A1,* **B**) *PSY-B1,* **C**) *ZDS-A1* and **D**) *LPX-B1.1*, averaged across water regimes. Violin plots with embedded boxplots are shown; points represent emmeans. *Different letters indicate significant differences according to Tukey’s HSD test* (*P < 0.05*)

The effect of allelic variation at each carotenoid biosynthesis gene was assessed by analysis of variance (Table 2). Significant effects on yellow pigment content were detected for *PSY-A1* and *PSY-B1*, with *PSY-A1* explaining the largest proportion of phenotypic variance (17.9%), followed by *PSY-B1* (14.8%). The *ZDS-A1* gene also showed a significant influence on that trait but to a lesser extent. Differences in mean yellow pigment content among alleles of these genes are shown in Fig. 3. For *PSY-A1* gene, the highest mean was associated with the *Psy-A1o* allele with 10.6 mg/kg, followed by *Psy-A1l* with a mean value of 9.0 mg/kg, and *Psy-A1a* with 6.4 mg/kg. With regards to *PSY-B1* gene, genotypes with the *Psy-B1n* allele showed higher carotenoid content, with a mean value of 10.2 mg/kg, compared to those with the *Psy-B1o* allele, with 7.2 mg/kg. Finally, for the *ZDS-A1* gene, the *TdZds-A1.2* allele was associated with higher pigment content, reaching 9.5 mg/kg in comparison with *TdZds-A1.1* allele, with a mean value of 7.8 mg/kg.

**Table 2:** Analysis of variance (ANOVA) for genes related to the synthesis and degradation of carotenoids in 46 durum wheat cultivars tested under full irrigation and drought conditions

Carotenoid synthesis genes		
Variation sources	d.f.	S.S.YPC (%)
*PSY-A1*	2	306.8^***^ (17.9)
*PSY-B1*	1	253.2^***^ (14.8)
*PDS-B1*	1	6.6 (0.4)
*ZDS-A1*	1	26.5^**^ (1.5)
Total	269	1714.0
*Carotenoid degradation genes*		
Variation sources	d.f.	S.S.YPL (%)
*LPX-A3*	1	139.8 (0.59)
*LPX-B1*	2	9724.1^***^ (41.1)
Total	266	23689.1

d.f.: degree of freedom; SS: sum of squares; YPC: yellow pigment content; YPL: yellow pigment loss. Values are significant for *P < 0.05, **P < 0.01, ***P < 0.001.

The influence of each gene involved in carotenoid degradation was assessed by an analysis of variance (Table 2). The results pointed out that *LPX-B1.1* was highly significant, explaining 41.0% of the variance. In contrast, *LPX-A3* had no significant effect. The lowest average degradation percentage was observed in cultivars carrying the *Lpx-B1.1c* allele, with a mean value of 5.9%. Cultivars exhibiting the *Lpx-B1.1a* allele showed an intermediate mean degradation percentage of 17.1%. The highest degradation was observed in the only cultivar carrying the *Lpx-B1.1b* allele, cv. Tomouh, with a mean value of 37.5%.

## Discussion

The bright yellow colour of semolina is a key quality trait throughout the pasta supply chain, from producers to consumers, influencing both visual appeal and market value (Borrelli et al. 2003; Randhawa et al. 2013). Furthermore, the yellow pigmentation of semolina and pasta – primarily due to lutein – is not only an aesthetic attribute but also provides nutraceutical benefits, particularly for eye health and reduction of oxidative stress (Mitra et al., 2021). Increasing restrictions on artificial colorants in pasta production have heightened the importance of breeding programs focused on improving this trait (Sissons 2008).

The collection evaluated in this study displayed a wide range of YPC, with the highest values being more than fivefold higher than the lowest, reflecting the diversity available in modern durum wheat germplasm worldwide. The collection included cultivars from breeding programs with differing target markets and breeding objectives, including landmark varieties recognized for high industrial quality or agronomic performance. The upper range of YPC included cultivars from “Desert Durum”, Australian and Italian breeding programs which have historically emphasized genetic improvement of quality, particularly yellow colour. In contrast, the lower YPC values were mainly observed in older North African and South Asian cultivars bred primarily for yield to enhance local food security, with little selection emphasis on YPC. While YPC reflected the priority given by different breeding programs to this trait, this was not the case for YPL, a trait that has been largely overlooked in breeding programs until recently. The results of the present study indicate that YPL is independent of YPC, as expected given their independent genetic bases of both traits.

Several studies have examined the genetic basis of YPC, concluding that it is controlled by multiple genes with additive effects and is influenced by environmental conditions (Schulthess et al. 2013). However, the role of environmental factors has often been underestimated due to the strong genetic control of the trait. Climate change may compromise various aspects of the global food supply chain such – including food quality, pricing, and distribution (Abbass et al. 2022). Altered precipitation patterns, increased frequency of droughts and floods, and reduced aquifer recharge affect water availability, making it less predictable. Given that durum wheat is cultivated under both irrigated and rainfed systems, it is essential to understand how contrasting environments could influence carotenoid synthesis and degradation. In the present study, initial carotenoid content was highly genotype-dependent, but cultivars responded differently under irrigated and drought conditions. In most cases (83% of cultivars), YPC was lower under drought stress. These results highlight the importance of water availability for optimal carotenoid accumulation, with higher concentrations generally achieved under irrigation. Our findings, which show higher pigment concentrations under irrigation, align with those reported by Güler et al. (2003), whose study emphasizes the critical importance of water availability, especially during vegetative stages. Carotenoids such as violaxanthin and neoxanthin are precursors of abscisic acid (ABA). Under drought conditions, plants increase ABA synthesis to regulate stomatal closure and reduce water loss. The lower carotenoid content obtained under drought conditions in this study could be explained by a deviation in the biosynthetic pathway toward ABA production (Cutler et al., 1999).

The role of LPX in durum wheat end products is well documented. However, difficulties and costs associated with measuring LPX activity or colour degradation during milling and pasta processing have historically limited its incorporation into breeding pipelines (Clarke et al., 2001). Fu et al. (2013) developed a micro-scale method that was, used in this study, which allows measurement of YPC while simulating pigment degradation (YPL) during pasta processing. In this study, carotenoid degradation was higher under drought than under irrigation. LPX contributes to the degradation of polyunsaturated fatty acids, helping to regulate the accumulation of reactive oxygen species (ROS) under stress conditions and protect cell membranes. Under drought stress, LPX can also contribute to jasmonic acid synthesis, regulating defense responses such as stomatal closure to reduce water loss, accumulation of defense proteins against oxidative stress, and ROS-mediated stress signalling (Sallam et al. 2019). Another possible explanation is that drought reduces grain filling efficiency, increasing the bran-to-endosperm ratio and potentially raising relative LPX concentration due to its localization in bran (Leenhardt et al. 2006). Together, these factors may explain the higher degradation observed under drought. It should be noted that the two water regimes in this study were managed with corresponding differences in nitrogen inputs. Consequently, the observed differences in YPC and YPL may reflect this combined management. However, while nitrogen availability can influence pigment content, research in durum wheat shows that variation in nitrogen dose above sufficiency levels has minimal impact on yellow pigment traits compared to water availability (Garrido-Lestache et al. 2005). In this study, nitrogen was applied at levels sufficient to avoid deficiency in both regimes. Therefore, the differences may be attributable primarily to the contrasting water availability. These results indicate that breeding for higher pigment content in drought-prone environments should consider both YPC and YPL under water-limited conditions to ensure accurate selection.

Carotenoid content is a quantitative trait regulated by multiple genes with additive effects. Its high heritability has facilitated genetic improvement, and mapping studies have identified quantitative trait loci (QTL) on numerous wheat chromosomes (Colasuonno et al. 2019). The most influential gene on YPC and yellow index (YI) is *PSY*, which is essential for carotenoid accumulation in kernels (Gallagher et al. 2004). Although the effect of *PSY* allelic variation depends on genetic background, previous studies found that *PSY-A1* explains medium to high phenotypic variation, while *PSY-B1* has low to medium effects (Campos et al. 2016; Parada et al. 2020). In our study, *PSY-A1* explained 17.9% of phenotypic variation, while *PSY-B1* explained 14.8%. The allele associated with the highest carotenoid content was *Psy-A1o* (10.6 mg carotenoids/kg flour), followed by *Psy-A1l* (9.0 mg/kg) and *Psy-A1a* (6.4 mg/kg). Previous studies also identified *Psy-A1a* as the allele associated with the lowest carotenoid content (Campos et al. 2016; Parada et al., 2020). However, there was no consensus on whether *Psy-A1l* or *Psy-A1o* was most beneficial. Here, *Psy-A1o* was clearly associated with the highest pigment content. For *PSY-B1*, our results agree with previous reports, being the *Psy-B1n* allele associated with a higher pigmentation (10.2 mg carotenoids/kg flour) compared to *Psy-B1o* (7.2 mg/kg) (Zhang et al., 2008).

Rasheed et al. (2016) reported a phytoene desaturase (*PDS*) gene with the *Pds-B1a* allele associated with a higher carotenoid content. In this study only the Mexican cultivar Atil C2000, presented this allele, so its effect could not be determined because of its very low frequency in the set evaluated. Finally, regarding *ZDS-A1* gene, cultivars carrying the *TdZds-A1.2* allele with a mean value of 9.5 mg/kg, were found to produce significantly higher pigment levels than those with the *TdZds-A1.1* allele with 7.8 mg/kg, consistent with the findings of Pasten et al. (2021). Most breeding programs have traditionally focused selection on the *PSY-A1* locus, considered to be the primary determinant of the carotenoid content. However, there are opportunities for further improvement capitalizing on the accumulation of favourable alleles of other genes, such as *PSY-B1* or *ZDS-A1*.

Carrera et al. (2007) mapped the lipoxygenases LPX-A3 and LPX-B1.1 in the Kofa ⋅ UC1113 population. The *LPX-B1.1c* allele was found to be important as it is associated with the loss of lipoxygenase function, preventing yellow pigment degradation. Consequently, most research has focused on *LPX-B1.1* gene rather than *LPX-A3*. In this study, no significant differences in YPL were associated with *LPX-A3* allelic variation. In contrast, strong differences in YPL in related to *LPX-B1.1* alleles were observed. The highest degradation corresponded to the *Lpx-B1.1b*, with a percentage of degradation of 37.5%, followed by *Lpx-B1.1a* allele with 17.1% colour loss and the null allele *Lpx-B1.1c*, with 5.9%. These results align with Verlotta et al. (2010) who reported high, intermediate and low levels of degradation for *Lpx-B1.1b*, *Lpx-B1.1a* and *Lpx-B1.1c*, respectively.

In conclusion, this study suggests that irrigation may favour carotenoid accumulation in durum wheat, while drought tends to increase pigment degradation, possibly due to metabolic shifts toward ABA biosynthesis, increased lipoxygenase activity, or increased bran-to-endosperm ratio. Some of the most important cultivars worldwide were characterized for their allelic variation related to yellow colour, revealing limited targeted selection for these alleles to date. Genetically, *PSY-A1* had the strongest effect in our dataset on YPC, with *Psy-A1o* allele associated with higher carotenoid accumulation. Colour degradation was primarily influenced by *LPX-B1.1*. The results of this study suggest that the combination of the *Psy-A1o*,* Psy-B1n*,* TdZds-A1.2 and Lpx-B1.1c* alleles could potentially enhance and maintain yellow pigmentation in semolina and pasta. These findings provide useful guidance for selecting cultivars with greater pigment stability; however, some alleles were represented by only a few cultivars, and further validation in larger, more diverse populations under varying environments is recommended to optimize their use in breeding programs.

## Supplementary Information

Below is the link to the electronic supplementary material.

Supplementary Material 1 (XLSX 44.6 KB )

Supplementary Material 2 (DOCX 14.4 KB)

## Data Availability

All data related to this study are available in Supplementary Table S1.
