# Supplementary material for: Allelic variation of carotenoid biosynthesis and degradation genes across worldwide commercial durum wheat (Triticum turgidum ssp. durum) varieties under contrasting water regimes
Source: Mol Breed. 2026 Feb 18;46(3):19. doi: 10.1007/s11032-026-01641-0 (PMC12917035; doi:10.1007/s11032-026-01641-0)
Supplement: Supplementary file 2 — Supplementary Material 2 (DOCX 14.4 KB) [file 11032_2026_1641_MOESM2_ESM.docx]

**Supplementary Material**

| **Table S2**. Analysis of Variance for YPC and YPL measured in a set of 46 durum wheat cultivars grown under 2 water regimes at CENEB, Cd. Obregon Sonora in 2015. | | | | |
| --- | --- | --- | --- | --- |
| Variation sources | d.f. | S.S.YPC (mg/Kg) | d.f. | S.S. YPL (%) |
| Cultivar | 45 | 1880.76 (95.88) ^***^ | 45 | 18418.94 (77.46) ^***^ |
| Water regime | 1 | 29.03 (1.48) ^***^ | 1 | 267.15 (1.12) ^***^ |
| Cultivar × Water regime | 45 | 43.36 (2.21) ^***^ | 45 | 4450.37 (18.72) ^***^ |
| Error | 178 | 8.41 (0.43) | 176 | 642.23 (2.70) |
| d.f.: degree of freedom; SS: Sum of squares; YPC (mg/Kg): Yellow Pigment Content; YPL (%): Yellow Pigment Loss. Significance levels: ^***^ *p* < 0.001, ^**^ *p* < 0.01, ^*^ *p* < 0.05 | | | | |
